# Immunolocalization of chromosome-associated proteins in plants – principles and applications

**DOI:** 10.1186/s40529-014-0063-5

**Published:** 2014-08-05

**Authors:** Cristina Maria Pinto de Paula, Vânia Helena Techio

**Affiliations:** Department of Biology, Federal University of Lavras, Lavras, Zip code 372000-000 Minas Gerais State Brazil

**Keywords:** Antibodies, Mitosis, Meiosis, Molecular cytogenetics, Epigenetics

## Abstract

The use of the immunolocalization technique combined with cytogenetic and epigenetic studies is an indispensable tool and has contributed significantly to the analysis of the structure and function of chromosomes, since it can provide information about the spatial or temporal distribution of a given protein in the nucleus and chromosomes. Several chromosome-associated proteins in plant cells have already been identified by immunolocalization, such as histone and non-histone proteins and cell division-related protein (mitosis and meiosis). The principle of the immunolocalization technique in plants basically involves fixation and permeabilization of cells, the use of monoclonal or polyclonal antibodies attached to a signaling molecule, usually a fluorochrome and detection of the target molecule by using an epifluorescence microscope.

## Introduction

Considerable advances in plant biology have been accompanied by the use of microscopy of higher resolution and improvement of the detection limit of analyses through use of specific antibodies and fluorochromes, that provide an interaction between knowledge of cell biology, classical cytogenetics and molecular cytogenetics. From the development of molecular cytogenetic techniques, understanding the organization of genomes had a large impact on taxonomic and evolutionary studies (Fedak and Kim [2008]; Figueroa and Bass [2010]), in breeding and genetic engineering (Seijo et al. [2010]).

Recent research on the architecture of the interphase nucleus and chromatin remodeling, mediated primarily by changes in histones, has prospected the era of epigenetics and epigenomics. In this new scientific background, the use of the technique of immunolocalization in chromosome analysis has become an indispensable tool for understanding the organization and expression of the genome of species. As a result of this interaction, significant improvements were obtained for the analysis of chromosome structure and function, as it can provide information about the spatial and temporal distribution of a given protein in the nucleus and chromosomes.

These analyses have been widely applied in recent decades to study and locate proteins in cells or cell populations and comprise the use of specific antibodies to the protein of interest (Hoshi et al. [2008]). In the context of chromosomal studies, advances are more recent, but have already made possible to identify several chromosome-associated proteins in plant cells, including histones (Fuchs et al. [1998]), non-histones (Stepinski [2009]), proteins associated with mitosis (Caperta et al. [2006]) and with meiosis (Franklin et al. [1999]; Armstrong et al. [2002]; Qiao et al. [2011]).

Given the importance of the topic and potential use, this review aims to present the principles of the immunolocalization technique and its application in the detection of proteins associated with chromosomes in plants.

## Review

### Principles of immunolocalization

The application of the immunolocalization technique was facilitated through the development of fluorochrome-labeled antibodies, in the pioneering work of Albert Coons (Coons et al. [1941]), thus allowing visualization of specific molecules in a cell. Therefore, it has been possible to observe the spatial or temporal distribution of a given protein in the whole cell, in the nucleus, in specific chromosome or organelles.

The procedures started to be used in plants from the experiences observed in animal cells. One of the first descriptions of the use of immunolocalization in plant cytology occurred in 1970, with the location of antigens of the cell wall in pollen (Knox [1970]) and proteins of cotyledons (Graham and Gunning [1970]). Currently, the immunolocalization has allowed, among other studies, the identification of chromosomal proteins, that is, the study of how the activity of these proteins is regulated during cell divisions (Seijo et al. [2010]; Houben et al. [2013]), and also the assessment of post-translational changes in histones, which are reflected in the chromatin structure, known as epigenetic regulation (Chen et al. [2010]).

The immunolocalization technique is based on the principles of antigen-antibody reactions to find highly specific molecules in a cell or tissue. The common reagent for immunolocalization are polyclonal or monoclonal antibodies, both produced in animals such as rabbits, goats, rats, mice, among others, by means of specific procedures (Boenisch [2009]).

Polyclonal antibodies are a heterogeneous mixture of antibodies that bind to different epitopes of the same protein. Monoclonal antibodies are homogeneous populations, more specific for binding to only one epitope of the protein (Boenisch [2009]).

There are many commercially available antibodies, some of universal use for chromosomal proteins, such as anti-tubulin (α, β and γ) or for modified DNA regions, such as the anti-5-methylcytosine (Guerra [2012]; Birchler and Han [2013]). Other antibodies are specific for a species or group of species, e.g., phosphorylated H2AThr120, which is used for cytological detection of centromeric chromatin of plants (Dong and Han [2012]; Demidov et al. [2014]).

In addition to factors related to the type of antibody, storage conditions and dilution of antibodies, which are usually indicated by the manufacturers, the immunolocalization reactions must take into account factors like affinity, avidity and cross-reactivity.

Affinity refers to the interaction strength between the antigen epitope and the paratope of an antibody. Similarly, avidity is the term used to describe the overall strength of the interactions between the antigen and the various antibodies that recognize it (Steward and Steensgaard [1983]; Boenisch [2009]). High affinity antigen-antibody means greater tendency to hold them together (Boenisch [2009]). However, factors such as high salt concentration, high temperature and low pH during the washing cycles applied in the technique, may result in the dissociation of the antigen-antibody complex.

Cross-reactivity occurs when an antibody reacts with two or more antigens, or when an antigen reacts with several different antibodies. In this case there is a sharing of at least one common epitope among the various antigens (Boenisch [2009]).

For detection of proteins by immunolocalization, the cells must be fixed and permeabilized to facilitate the access of antibodies to the cytoplasm and nucleus. Fixation of the nucleus or chromosomes must preserve the arrangement of molecules as they occur in vivo, and treatment should not affect the cytological integrity of the material to be analyzed. In general, aldehyde fixatives are used, which make a link between proteins, nucleic acids and phospholipids, thus forming a network which retains molecules in their original position. Formaldehyde, paraformaldehyde and glutaraldehyde are the most frequently used in immunolocalization (Marttila and Santén [2007]).

There are some cases in which acid fixatives can be effective to the meiosis, for example, Carnoy (ethyl alcohol: acetic acid 3:1) (Chelysheva et al. [2010], [2013]). In this last case, the fixation is associated with microwave procedures. This method combines a strong fixation to preserve the chromosomal structures, acetic acid spreading to remove the cytoplasm and treatment in microwave (850 W) to increase the access of the antibody to the protein.

Another possibility for meiosis is use of the methanol/acetone fixative, which extracts the lipids and rapidly dehydrates the cell, thus disrupting hydrophobic interactions and resulting in aggregation and precipitation of proteins. This fixative preserves cell architecture, but can result in reduced antigenicity of some proteins. Nevertheless, it can also allow better access of the antibody to certain antigens without requiring cell permeabilization (Yang et al. [2013]).

Permeabilization is usually carried out by incubation with detergents or organic solvents, which solubilize or remove lipids from the plasma membrane and thus allow these antibodies to have access to the structure of interest. The most commonly used permeabilizing agents include Triton X-100, Tween 20 and also Lipsol. These are hydrophilic nonionic detergents, considered mild surfactants, in other words they break lipid-protein and lipid-lipid interactions, but not protein-protein bonds, maintaining their active form (Johnson [2013]).

In the case of plant cells, it is necessary to overcome the barrier of the cell wall, so that permeabilization usually requires a prior cell wall digestion using enzyme cocktails containing pectinase, cellulase, pectoliase and others, whose concentration and exposure time are adjusted according to the species analyzed.

The usual procedure for plants is indirect immunolocalization. This technique includes the use of at least two antibodies: the primary which recognizes and binds to epitopes of the antigen (protein) to be located, and the secondary, often conjugated to a fluorochrome, which recognizes the corresponding primary antibody, the reason why the procedure is also referred to immunofluorescence. Alternatively, gold conjugated secondary antibodies can be used, in this case, the visualization is performed in a transmission electron microscope and the procedure is called immunogold (Holgate et al. [1983]; Marttila and Santén [2007]). The direct method employs only one antibody linked to a fluorochrome. The advantage of indirect labeling is the amplification of the signal obtained, since several secondary antibodies may bind to a single primary antibody (Brown and Lemmon [1995]; Marttila and Santén [2007]).

Fluorochromes are chemicals that, upon excitation at a specific wave length, fluoresce at another wavelength, and the signal is observed with an epifluorescence microscope (Brown and Lemmon [1995]). Fluorescein (FITC) is the fluorochrome most used in immunolocalization, it can be excited by UV light (wavelength below 400 nm) or blue light (490 nm) and emits green light (500–550 nm), as well as Rhodamine (TRITC) and Texas red, fluorochromes which are excited between 520 and 590 nm and emit red light (550–620 nm) (McNamara [2006]). There are other types of fluorochromes with higher fluorescence intensity and photostability, compared with those aforementioned and may also be used in immunolocalization such as Dylight, Alexa Fluor (Jensen [2012]) and quantum dots, the latter a class of inorganic fluorochromes made up of nanoscale crystals of a semiconductor material (Ioannou et al. [2009]).

An alternative methodology proposed by (Wang [2013]) analyzes meiotic chromosomes by three-dimensional structured illumination microscopy (3D-SIM) using high resolution images and can be applied for the localization of proteins or fine structures by immunofluorescence. The protocol (Acrylamide sandwich) was developed aimed at better preservation of 3D chromosome structure and spatial organization of the nucleus; in turn the purpose of 3D-SIM is to illuminate a sample with a light distribution pattern produced by a diffraction grating. With this technique, several optical sections are captured and a 3D image is reconstructed by computer analysis.

### Major chromosome-associated proteins localized in plants

#### Study of non-histone protein

A large number of studies have already identified several proteins associated with chromosomes in plant cells so far. The study of the behavior of these proteins provide information about the function of chromosomes, since part of the control of the genetic information encoded by DNA depends on interactions with proteins. The immunolocalization of chromosomal proteins in plants has been widely used for different purposes in cytogenetic and epigenetic studies such as identification of proteins that are directly or indirectly involved with mitosis and meiosis and associated with chromatin modeling.

The proper chromosome condensation during mitosis and meiosis is essential for the correct segregation of the genomic information contained in the DNA. Besides histones, other non-histone proteins are also involved in the process of condensation and cohesion, such as a topoisomerase II, condensins and cohesin (Moser and Swedlow [2011]).

Cohesin complex proteins are essential for sister chromatid cohesion and proper chromosome segregation during mitosis and meiosis. Cohesin proteins are also components of axial elements (AEs) and lateral elements (LEs) of the synaptonemal complex during meiosis. The cytological behavior of four cohesin proteins (SMC1, SMC3, SCC and REC8/SYN1), assessed by immunofluorescence during prophase I in tomato microsporocyte revealed that the four cohesins are distributed unevenly and are not co-located along the AE/LEs in diplotene (Qiao et al. [2011]). Nevertheless, based on current models of the cohesin complex, these proteins must be present at the same time and place in equivalent amounts. These results indicate that cohesin proteins studied can form different complexes and/or perform additional functions during meiosis in plants.

The success of meiosis depends on the regulation of several cellular processes that ensure proper chromosome segregation. By employing the immunolocalization technique it is possible to visualize a large number of proteins related to different aspects of meiosis, including sister chromatid cohesion, synapsis, recombination, and chromosome segregation (Yang et al. [2013]).

A number of proteins involved in the pairing and recombination are expressed during meiosis, including RAD51 and MLH1. Studies with maize have shown that RAD51 is involved in the homology search during chromosomal pairing and recombination. Through immunolocalization, it was observed, in zygotene, 500 RAD51 signals per nucleus, clearly associated with the partially paired and paired chromosomes, which is consistent with its role in the homology search. In pachytene, the number of RAD51 signals decreased, ranging from 7 to 22 per nucleus, with the largest number corresponding approximately to the number of chiasmata found in maize, thus coinciding with the expected number of recombination during meiosis (Franklin et al. [1999]).

Likewise, RAD51 protein in *Arabidopsis* is related to homology search and meiotic recombination (Kurzbauer et al. [2012]). *Atrad51*-*2* mutants express low amounts of the RAD51 protein, meiosis with partial synapsis and 51% of bivalents between non-homologous chromosomes, thus demonstrating that RAD51 is required for effective chromosome pairing (Pradillo et al. [2012]).

Similar studies were developed with *Lily*, being detected the presence of RAD51 and LIM15 proteins as discrete signals in leptotene and zygotene. This localization suggests that meiotic recombination is initiated in leptotene with the cooperation of these two proteins and remains in zygotene. The location of the signals on chromosomes or in their vicinity suggests that these proteins must also act in the pairing of homologous chromosomes in this species (Terasawa et al. [1995]; Anderson et al. [1997]).

The ASY1 gene is essential for synapsis in homologous chromosomes (Caryl et al. [2000]). Studies using specific antibodies against the ASY1 protein were applied to investigate the temporal expression and localization of this protein in *Arabidopsis thaliana* (L.) Heynh. Detection of the immuno signal associated with electron microscopy data showed that ASY1 is not a component of the synaptonemal complex, but is associated with the axial/lateral elements of meiotic chromosomes. The authors suggest that this protein possibly defines the regions of chromatin associated with the development of the synaptonemal complex structure (Armstrong et al. [2002]).

Cytoskeleton microtubules play a crucial role during the cell cycle and meiosis. The use of antimitotic substances, such as colchicine, promotes depolymerization of microtubules and prevents the formation of spindle fibers, this methodology is used to block cell cycle progression and thus induce polyploidy (Pereira et al. [2012]). Through immunolocalization of alpha-tubulin, (Caperta et al. [2006]) analyzed the effect of different colchicine concentrations on spindle fibers in *Secale cereale* L. At a low concentration of colchicine (0.5 mM) most of the cells showed arrays of discontinuous microtubule or no microtubule were detected at C-metaphase. Cells exposed to high concentrations (5.0 mM) showed a different effect, the C- metaphase presented fibrous and branched cortical filaments that allowed reconstitution of 4C nuclei and cell cycle progression. These results demonstrate the contrasting and opposite effects at different concentrations of colchicine.

#### Study of histone modifications

Histones form another group of proteins associated with eukaryotic DNA, comprising the nucleosome, which consists of an octamer of the four major histones (H3, H4, H2A, H2B) (Luger et al. [1997]; Kouzarides [2007]).

Each histone that comprises the nucleosome has a terminal amino acid chain, called N-terminal tail, which is subjected to various post-translational modifications (Kouzarides [2007]; Jin et al. [2008]). These epigenetic marks affect the structure and function of chromatin, thereby regulating gene expression and can be identified by immunolocalization (Bannister and Kouzarides [2011]).

There are at least eight different types of alterations in histones, standing out acetylation, methylation and phosphorylation, which are the most studied (Kouzarides [2007]; Chen et al. [2010]).

Plant chromosomes usually exhibit a lower amount of acetylated histones in heterochromatin and often form significant markings in the nucleolar organizing region (NOR), which allows a correlation with active gene transcription. In *Vicia faba* L., antibodies against acetylated H4 at lysine 5, 8 and 12 labeled the entire chromosome complement, with the exception of large blocks of heterochromatin (Fuchs et al. [1998]), since the NOR was the most strongly acetylated of all lysine residues investigated (Houben et al. [1996]).

One of the most studied post-translational modification is the phosphorylation of histone H3, which seems to be decisive for cell cycle in relation to the chromosome condensation and segregation, activation of apoptosis, transcription and repair of DNA damage (Houben et al. [2007]). In plants, phosphorylation of histone H3 at serine 10 (H3S10ph) and 28 (H3S28ph) is restricted to the pericentromeric region in mitosis (Germand et al. [2003]). In meiosis, the chromosomes are phosphorylated along the entire arm during the first division, however, the phosphorylation is restricted to the pericentromeric region during the second division (Manzanero et al. [2000]).

In studies with forage grasses Paula et al. ([2013]) evaluated the phosphorylation pattern of H3S10 in diploid and tetraploid genotypes of *Brachiaria* (Trin.) Griseb. species in order to investigate the dynamics of this post-translational modification and correlate it with its regulatory function during mitosis and meiosis. In meiosis, the chromosomes are phosphorylated at H3S10 along its entire length during the first division; in the second division, phosphorylation is restricted to the pericentromeric region, as well as in mitosis. Alltogether, these observations indicated that H3S10ph is necessary for the maintenance of sister chromatid cohesion in mitosis and meiosis.

Phosphorylation of threonine in histone H3 in *Secale cereale* L. and threonine 11 in H3 in *Vicia faba* L. was observed throughout the chromosome during mitosis and meiosis. Phosphorylation began in prophase and ended in telophase, thus correlating with chromosome condensation (Houben et al. [2005], [2007]).

By means of immunolocalization, it was possible to detect that methylation of some isoforms of histones in plants may be associated with heterochromatin (H3K9, H3K27 and H4K20) and also euchromatin (H3K4, H3K36 and H3K79) (Fuchs et al. [2006]).

Different types of chromatin associated with histone modifications were analyzed by immunolocalization in *Costus spiralis*. Antibodies against components characteristic of histones of euchromatin (acetylated H4 histone at lysine 5 - H4K5ac and dimethylated H3 histone at lysine 4 - H3K4me2) and heterochromatin (dimethylated H3 histone at lysine 9 - H3K9me2) were used to characterize the centromeric chromatin during meiosis. The centromeric region was highly enriched only with H4k5ac and only in pachytene. Still in pachytene, the terminal decondensed euchromatin of chromosome arms were labeled with H4K5ac and H3K4me2, whereas the condensed proximal region was labeled with H3K9me2 (Feitoza and Guerra [2011a]).

In plant species *Costus spiralis* and *Eleutherine bulbosa* during mitosis, labeling with antibodies against components characteristic of histones of euchromatin were observed in the regions of late condensation while the components of heterochromatin was more intense in the early condensation chromatin. These data indicate that there are different chromatin domains in these species, the late condensing prophase chromatin localized on the distal region and strongly enriched in H4K5ac and H3K4me2 and early condensing chromatin, localized on the proximal chromosome region in *C. spiralis* and mainly in the larger pair I of *E. bulbosa* (Feitoza and Guerra [2011b]).

Intraspecific hybrids and parental acessions (Col-0 and C24) of *Arabidopsis thaliana* were used as a model to investigate the relationship between changes in DNA methylation, chromatin structure, endopolyploidy and gene expression in heterotic genotypes. Through immunolocalization, the distribution of methylation of histone H3 (H3K27me3, H3K4me2 and H3K9me2) was compared between parental and hybrid plants. For all modifications of the chromatin analyzed, no clear difference was found in the distribution and intensity of signals between Col-0, C24 and their reciprocal hybrids, revealing that the parental pattern profile was kept in the hybrid progeny (Moghaddam et al. [2010]).

Besides that, the correlation between the distribution of histone H3 methylated at lysine 4 (typical of transcriptionally active euchromatin) and lysine 9 (inactive heterochromatin) was analyzed in relation to genome size for 24 species. In species with small genome, strong H3K9me was restricted to constitutive heterochromatin, whereas species with large genomes exhibited uniform distribution of this modification. Unlike and independent of the genome size, H3k4me was enriched in euchromatin of all species. The authors concluded that large genomes with large amounts of dispersed repetitive sequences (mainly retroelements) silence these sequences through epigenetic modifications such as H3K9me (Houben et al. [2003]).

Centromeres are responsible for several important events in meiosis and mitosis. In contrast to the telomeres, centromeres do not have highly conserved DNA sequence and are determined by epigenetic modifications (Houben and Schubert [2003]). In eukaryotes, the centromeres have a variant of histone H3, the CENH3 (CENPA in humans) which determines the chromosomal position of kinetochore assembly, forming a bond between the centromeric DNA and the kinetochore (Blower et al. [2002]). Thus, the anti-CENH3 antibody has been used in studies related to the centromeric regions in plants like corn (Jin et al. [2005]; Zhang et al. [2005]), wheat (Zhang et al. [2010]) and rye (Houben et al. [2011]).

Recently, Demidov et al. ([2014]) evidenced that the antibody against phosphorylated histone H2A at threonine 120 can be used as a universal maker for the cytological detection of centromeres in species of monocentric and holocentric plants. These results were obtained from studies on 20 different species of mono- and eudicots.

Although histones and their modifications are highly conserved, their distribution in chromosomes, observed by immunolocalization, showed a variation over the differentiation and cell cycle processes, as well as within and between groups of eukaryotes (Hans and Dimitrov [2001]).

## Conclusion

Immunolocalization is an important and efficient tool in studies involving the understanding of genome organization and analysis of the structure and function of chromosomes. Furthermore, immunolocalization studies allowed the understanding of epigenetic mechanisms involved in chromatin modeling and modifications related to different states of condensation, such as transcriptional activity and silencing.
